# Measure of ^90^Y-glass microspheres residue post-TARE using PET/CT and potential impact on tumor absorbed dose in comparison ^99m^Tc-MAA SPECT/CT dosimetry

**DOI:** 10.1186/s41824-024-00214-8

**Published:** 2024-08-26

**Authors:** Sarah Boughdad, Rafael Duran, John O. Prior, Michael da Mota, Mélanie Mendes De Carvalho, Julien Costes, Maria Firsova, Silvano Gnesin, Niklaus Schaefer

**Affiliations:** 1 Department of Nuclear Medicine and Molecular Imaging, Lausanne University Hospital, Rue du Bugnon 46, 1011 Lausanne, Switzerland; 2 Department of Radiology, Lausanne University Hospital, Lausanne, Switzerland; 3 Faculty of Biology and Medicine, University of Lausanne, Lausanne, Switzerland; 4 Institute of Radiation Physics, Lausanne University Hospital and University of Lausanne, Lausanne, Switzerland

**Keywords:** ^90^Y-PET/CT, TARE, ^90^Y-glass microspheres, Residual activity, Dosimetry, TARE, Hepatocellular carcinoma

## Abstract

**Background:**

Transarterial radio-embolization (TARE) became a routine procedure for non-resectable liver tumor mainly hepatocellular carcinoma (HCC). Personalized dosimetry to the index lesion increased tumor response rate. However, there is no requirement to measure the precise activity injected during TARE. We measured ^90^Y-glass microspheres residue (^90^Y-Res) in the application system after TARE and assessed its potential impact on the tumor absorbed dose (AD) previously planned with ^99m^Tc MAA SPECT/CT.

**Methods:**

We measured ^90^Y-Res using PET/CT in all patients that underwent TARE using ^90^Y-glass-microspheres for non-resectable liver tumors over one year.

**Results:**

^90^Y-Res was measured in 34 patients (HCC n = 22) with 61 injections, 93.1 ± 94.6 MBq [2–437] that was 4.8 ± 3.5% [0.2–13.7] in comparison to the activity measured in the sealed TheraSphere™ vial (ρ = 0.697; *p* < 0.001).

**Conclusion:**

We reported an average of 5% ^90^Y-Res using PET/CT after TARE with the strongest association to the activity in the TheraSphere™ vial. Therefore, when a high ^90^Y-Res is suspected on the survey meter, a ^90^Y-PET/CT scan of ^90^Y-Res might be useful as a first step to estimate if the target lesion received the recommended AD, especially in HCC patients with borderline tumor dosimetry on the pre-treatment ^99m^Tc-MAA SPECT/CT.

## Introduction

Transarterial radio-embolization (TARE) has become a standard treatment in non-resectable primary liver tumors and liver-dominant liver metastases (HCC, Yang et al. 2020). A catheter placed in the hepatic artery feeding the tumor infuses ^90^Y-loaded glass or resin microspheres directly into the arterialized tumor bed with a good safety profile (Hong et al. 2021; Hilgard et al. 2010). Despite solid data in advanced disease, in early treatment settings the data is still limited, and several studies were negative on their primary endpoint (Hazel et al. 2016). Many different factors leading to negative trial results have been debated but the lack of personalized dosimetry was seen as the most relevant factor (Sposito and Mazzaferro 2018).

The recent recommendations mandated personalized dosimetry and established a relevant leap in the TARE procedure (Gnesin et al. 2016; Garin et al. 2020). Indeed, DOSISPHERE-01, a randomized multicenter open-label and phase 2 trial, investigated personalized dosimetry in HCC patients aiming at an absorbed dose (AD) of > 205 Gy on tumor lesions in comparison to the standard partition model aiming at 120 ± 20 Gy per tumor lesion. This trial showed significantly better response rate and survival outcomes for patients treated with a personalized dosimetry procedure without significant increased toxicity (Garin et al. 2021). Similarly, the recent TARGET study has shown that the implementation of a more robust, standardized dosimetric approach delivered more consistent results (Lam et al. 2022). There is much hope that future trials relying on standardized dosimetry might lead to optimal therapy planning, lower variation in dosing schemes and ultimately better trial results (Vilgrain et al. 2014; Chow et al. 2018; Mazzaferro et al. 2013).

Personalized dosimetry in TARE with ^90^Y-glass microspheres is routinely based on ^99m^Tc-macroaggregated albumin single-photon emission computerized tomography ^99m^Tc-MAA SPECT/CT to predict AD in Gray (Gy) to the index lesion, healthy perfused liver tissue and lungs with the accurate definition of the tumor volume and the lung shunt fraction being essentials (Busse et al. 2023). The prescribed administered therapeutic activity is fixed to reach a threshold AD to index lesions used as a predictor of tumor response (i.e., ≥ 205 Gy for HCC patients while limiting the absorbed dose to the healthy perfused liver tissue to a safe level (Garin et al. 2021; Chiesa et al. 2021). This concept underlines the need of a complete administration of the prescribed ^90^Y-microspheres activity to avoid under-dosing the tumor. Various reasons could lead to an incomplete activity administration such as a technical error with incomplete voiding of the dose vial, flow phenomenon’ of glass microspheres in catheter connections, adhesion of ^90^Y-microspheres inside the catheter wall, among others, underlining the importance of thorough flushing of the injection system (Caine et al. 2017). Thus, in addition to a precise measurement of the ^90^Y activity on the day of the TARE procedure with a dose calibrator fulfilling the national standard, an accurate measurement of the residual radioactive waste (^90^Y-Res) is crucial to determine the dose delivered to the patient (13). Drescher et al. reported a mean residual activity in the application device of 3.4% ± 1.7 post-TARE using ^90^Y-glass microsphere in 18 consecutives TARE procedures using PET/CT (Drescher et al. 2020). Ebbers et al. confirmed the robustness of using ^90^Y-PET/CT for the measurement of the residual activity after TARE with an excellent correlation (ρ = 0.99) to the activity measured with the dose calibrator which remains the most accurate measurement method (Ebbers et al. 2020). Yet, routine measurement of the precise activity injected during TARE is not standardized and was not reported in the latest recommendations of an international working group for ^90^Y-glass microspheres (Salem et al. 2023). Additionally, its potential impact on the dosimetry, that is the actual tumor AD, has not been reported. In this study, we chose to focus on ^99m^Tc-MAA SPECT/CT dosimetry as it has been reported in the literature that it reflected ^90^Y-microspheres distribution (Lambert et al. 2010; Garin et al. 2016) whereas the literature for quantitative tumor AD using post-therapy ^90^Y-bremsstrahlung SPECT/CT is limited (Kim et al. 2021). Thus, we thought that it would be interesting to retrospectively input the “^90^Y-Res correction” consisting of the activity in TheraSphere™ vial measured before TARE minus the residual activity of ^90^Y-glass microspheres (^90^Y-Res) measured on ^90^Y-PET/CT on the pre-treatment ^99m^Tc-MAA SPECT/CT distribution simulation to have an estimate of the tumor absorbed dose. We aimed to prospectively evaluate the amount of residual activity of ^90^Y-Res after TARE using ^90^Y-PET/CT for a better understanding of potential shortcomings in the administration of the full activity of ^90^Y-glass microspheres. We also investigated which clinical and technical parameters could affect the amount of residual activity not administered to the patient after TARE and looked at its potential impact on the perfused tumor absorbed dose planned on the pre-treatment ^99m^Tc-MAA SPECT/CT.

## Methods

We prospectively measured the ^90^Y-Res, assessed at the patient administration time, after each injection for consecutive patients that underwent TARE using ^90^Y-glass-microspheres in our institution for non-resectable liver tumors from February 2021 to February 2022. The setup for all TARE procedures was done in agreement with the manufacturer recommendations (TheraSphere™; BTG International, Farnham, United Kingdom).

### Personalized dosimetry

Personalized dosimetry planning was performed on ^99m^Tc-MAA SPECT/CT using the Simplicit^90^Y™ software. This software allows for a personalised patient dosimetry based on a multi-compartment approach to define tumor absorbed dose: the whole liver volume, the perfused volume, the tumor perfused volume and the healthy liver tissue perfused volume. In case of a radiation segmentectomy, a single-compartment approach was used. Thus, the absorbed dose to the healthy liver tissue within the perfused volume was not accounted for and we aimed when possible for an absorbed dose of 400 Gy to the perfused volume. The software allowed for the fusion of the attenuation corrected ^99m^Tc-MAA SPECT imaging with anatomical imaging to insure that the ^99m^Tc-MAA simulation distribution within the perfused volume correspond to an optimal ratio between the anatomical tumor and the surrounding healthy liver tissue. In our institution for more accuracy we used for anatomical imaging the intra-arterial CT done in the angiography suite at the arterial position for the injection of ^99m^Tc-MAA.

### ^90^Y-Res assessment

**Fig. 1:**
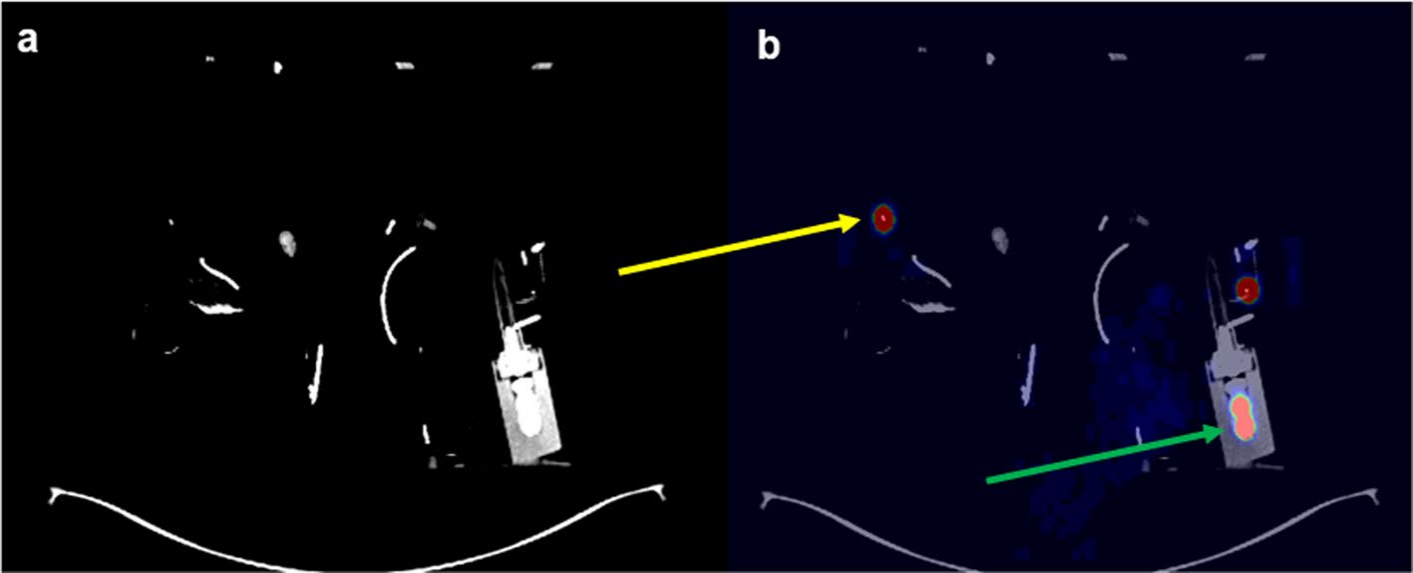
CT (**a**) and fused ^90^Y-PET/CT images (**b**) of the ^90^Y-res acquired post-TARE consisting of the administration device including the TheraSphere™ ^90^Y glass microspheres vial (green arrow) and the microcatheter used for the intra-arterial injection during the procedure (orange arrow)

^90^Y-Res was measured in the delivery tubing used during TARE, the TheraSphere™ vial and the microcatheter used for the procedure; all placed in a sealed plastic waste container (Fig. 1). We imaged the ^90^Y-Res after TARE on a digital SiPM Biograph VISION 600 PET/CT (Siemens Healhineers, Knoxville TS, USA): We performed static TOF PET, 20 min long, single bed, list-mode acquisitions covering an axial field of view of 26 cm. The PET measured ^90^Y-Res was decay corrected at the time of the patient administration. In patients who received multiple injections during TARE, ^90^Y-Res was measured in a separate box for each injection. PET/CT acquisition were reconstructed with the vendor iterative reconstruction algorithm (TrueX-HD) and using 2 iterations and 5 subsets and a post reconstruction Gaussian filter with FWHM = 4 mm. The in-plane reconstruction matrix size was 220 × 220 with a pixel spacing of 3.3 mm and a slice thickness of 1.4 mm: the attenuation correction was based on a low-dose CT scan (65 mAs, 120 keV, 0.8 pitch and 2.0 mm slice thickness). The scatter correction used an absolute scaling.

Patients also underwent post-TARE ^90^Y-bremsstrahlung SPECT/CT to confirm that the distribution of ^90^Y-microspheres was similar to the initial ^99m^Tc-MAA SPECT/CT. ^90^Y-bremsstrahlung SPECT/CT acquisition parameters were as follow: Flash 3D (3D OSEM: between 20 and 120 iterative updates and 2subsets) with 60 frames of 35 s, a gaussian filter between 0 and 10 mm FWHM, 256 × 256 matrix, 2.4 × 2.4 × 2.4 mm pixel size, 100–200 keV energy window and CT-based attenuation correction.

We assessed ^90^Y-Res for each injection site separately as several patients received multiple injections. For each injection procedure, we also assessed the percentage of the calibrated TheraSphere™ vial activity (measured before TARE in the activimeter by our radiopharmacist and decay corrected at the planned patient administration time) present in the respective ^90^Y-Res. We did not account for the potential time delay between the planned patient administration time and actual patient administration time as it was not significant in comparison to the longer half-life of the ^90^Y isotope. We also retrospectively inputed the “^90^Y-Res correction” consisting of the activity in TheraSphere™ vial measured before TARE minus the residual activity of ^90^Y-glass microspheres (^90^Y-Res) measured on ^90^Y-PET/CT on the pre-treatment ^99m^Tc-MAA SPECT/CT distribution simulation in order to have an estimate of the tumor absorbed dose using the Simplicit^90^Y™ software using the same procedure as previously described.

### Statistical analysis

We used *t*-test for dependent samples to compare the ^90^Y-glass microspheres administered activity based on ^99m^Tc-MAA SPECT/CT dosimetry and the activity measured in the sealed TheraSphere™ vial at the patient therapeutic administration time. Spearman correlation was used to assess correlation between ^90^Y-Res, the activity measured in the TheraSphere™ vial and the different target liver volumes (in cm^3^) defined on pre-treatment ^99m^Tc-MAA SPECT/CT: the perfused liver volume, the total perfused tumor volume and perfused healthy liver tissue obtained by Boolean subtraction of the perfused tumor volume from the perfused volume. Similar analyses were done with absorbed dose in Gy.

We used *t*-test for independent samples to compare ^90^Y-Res according to the type of TARE single-compartment versus multicompartment and the microcatheter position during TARE delivery: lobar versus non-lobar artery. ^90^Y-Res was compared as a function of the size of the microcatheter: Progreat 2.7Fr. microcatheter (P-cat, Terumo, Interventional Systems, Europe) versus ASAHI Masters Parkway Soft 1.98/2.8 F microcatheter (AP-cat, Asahi Intecc, Japan). To assess the impact of ^90^Y-Res on personalized dosimetry, we subtracted its activity to the activity of the TheraSphere™ vial and retrospectively applied this new activity value labelled “^90^Y-Res correction” to the dosimetry planning performed with the Simplicit^90^Y™ software on ^99m^Tc-MAA SPECT/CT. This new dosimetry assessment was expected to improve the estimation of perfused tumor AD, hence precisely defining patient’s specific dosimetry. We used *t*-test for dependent samples to compare absorbed dose between the initial and the “corrected dosimetry” previously defined for each compartment perfused volume and perfused tumor volume.

### Ethics

This study was done according to the ethical standards defined by the Helsinki declaration and its later amendments. Our local ethics committee that is the “ Commission cantonale d'éthique de la recherche sur l'être humain” (CER-VD) approved this retrospective study with informed consent waiver allowing the inclusion of all patients that did not explicitly refuse their consent as per the local legislation at the time of the study (registration number: CER-VD-2018-01513).

## Results

### ^90^Y-Res assessment

**Fig. 2:**
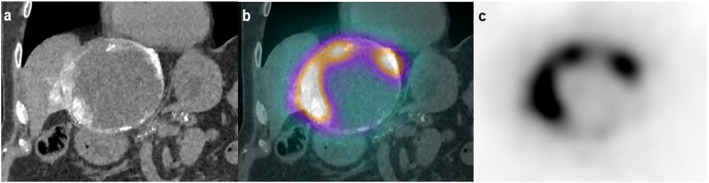
Voluminous HCC tumor with extensive necrosis and viable peripheral tumor in an 81-year-old patient treated with TARE: perfused volume of 81 cm^3^ estimated on pre-treatment ^99m^Tc-MAA SPECT/CT and a subsequent ^90^Y-Res of 11.6% on ^90^Y-PET/CT acquisition post-TARE; **a** coronal image of intra-arterial CT during TARE with soluble contrast media injected through the microcatheter, **b** coronal image of fused Bremsstrahlung ^90^Y-SPECT/ CT with intra-arterial CT during TARE and **c** coronal image of Bremsstrahlung ^90^Y-SPECT

**Fig. 3:**
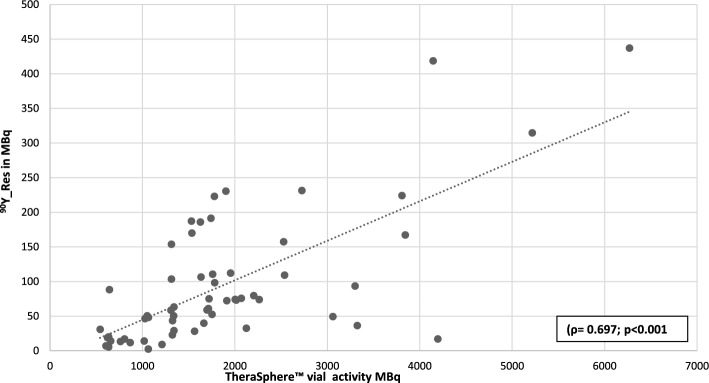
Correlation between ^90^Y-Res in MBq, measured using ^90^Y-PET/CT post-TARE and the activity in the sealed TheraSphere™ vial before TARE

We measured ^90^Y-Res in 34 patients (HCC n = 22, cholangiocarcinoma n = 4, metastases n = 8) for a total of 61 injections sites (Table 1, Fig. 2). The mean value ± 1SD for ^90^Y-Res was 93.1 ± 94.6 MBq [2–437] that is a mean 4.8 ± 3.5% [0.2–13.7] of the activity measured in the TheraSphere™ vial prior to the TARE. There was a significant difference between the intended ^90^Y-glass microspheres activity planned on the pre-treatment ^99m^Tc-MAA SPECT/CT and the activity measured in the sealed TheraSphere™ vial on the day of the TARE with significantly higher values in the latter (Table 2; *p* < 0.001). However, when ^90^Y-Res was subtracted from the activity measured in the sealed TheraSphere™ vial, there was no longer a significant difference with the intended activity planned on the pre-treatment ^99m^Tc-MAA SPECT/CT (Table 2; *p* = 0.59). There were significant and positive correlations between absolute value in MBq of ^90^Y-Res with the planned activity (ρ = 0.689; *p* < 0.001) and the activity measured on the TheraSphere™ vial (ρ = 0.697; *p* < 0.001, Fig. 3).

**Table 1:** Patients’ characteristics in terms of histological types and the number of TARE procedures and injections

	Histological type	Number of patients	Number of TARE procedures	Number of injections
Primary liver tumor	Hepatocellular carcinoma	22	25	36
	Cholangiocarcinoma	4	5	10
Secondary liver tumors	Breast	1	1	1
	Colorectal cancer	1	1	2
	Leiomyosarcoma	1	1	1
	Malignant adrenocortical cancer	1	2	2
	Neuroendocrine tumors	3	5	8
	Ocular melanoma	1	1	1
	Total	34 patients	41 procedures	61 injections

**Table 2:** Comparison of ^90^Y-glass microspheres activity planned on the pre-treatment ^99m^Tc-MAA SPECT/CT with the ordered ^90^Y-microspheres activity ^1^, the TheraSphere™ vial and when applying the “^90^Y-Res correction” ^2^ obtained after subtraction of ^90^Y-Res activity to ^90^Y activity in the TheraSphere™ vial

	Mean ^90^Y Activity ± SD in MBq	^90^Y Activity range in MBq	Difference with planned activity in %	Difference with planned activity *p* value
^90^Y activity planned on pre-treatment ^99m^Tc-MAA SPECT/CT ^1^	1713 ± 1121	[500–6000]	–	–
^90^Y activity in the TheraSphere™ vial	1781 ± 1182	[542–6269]	5.8 ± −3.7	< 0.001
^90^Y activity with “^90^Y-Res correction” ^2^	1752 ± 1107	[511–5932]	0.8 ± 4.3	0.59

### ^90^Y-Res and TARE procedure

We analyzed a total of 48 single-compartment treatment planned as radiation segmentectomy in this cohort and 13 multi-compartment treatments. There was no significant difference in the value of ^90^Y-Res between single or multicompartment treatments (mean 88.4 ± 96.8 MBq versus 116.8 ± 85.6 MBq and mean 4.8% ± 3.7 versus 6.4% ± 4; *p* = 0.34 and *p* = 0.20 respectively). Similarly, there was also no significant difference for the intended therapeutic activity as a function of the treatment type (*p* = 0.58). There were, however, significant differences between perfused volumes with significantly smaller volumes in patients that had a single-compartment TARE when compared to multicompartment treatments with mean 342 ± 368 cm^3^ versus 894 ± 513 cm^3^, respectively (*p* < 0.001). Similarly, AD was significantly higher when a radiation segmentectomy was planned for both the perfused volume and perfused tumor volume: mean AD 387 ± 264 Gy versus 148 ± 87 Gy and mean AD 548 ± 486 Gy versus 223 ± 80 Gy, respectively (*p* < 0.001).

### Parameters impacting ^90^Y-Res

There were 11 TARE injections at a lobar position and 50 injections at sub-lobar position including 35 injections at segmental an/or subsegmental levels. There was a significant difference in the activity of the ^90^Y-Res depending on the artery position for the delivery of ^90^Y-glass microspheres with a significantly higher ^90^Y-Res at a lobar position 205.6 ± 137 MBq versus 69.2 M ± 59.1 Bq for sub-lobar TARE or 6.5 ± 3.3% versus 4.6 ± 3.7% of the activity in the TheraSphere™ vial prior to the TARE for lobar and sublobar injections respectively (*p* = 0.008). Intended administered activity for lobar injection was also significantly higher with a mean value of 2986 ± 1551 MBq versus 1467 ± 767 MBq (*p* = 0.009) so as both perfused volume 911 ± 338 cm^3^ versus 338 ± 291 cm^3^ and perfused tumor volume 428 ± 306 cm^3^ versus 159 ± 175 cm^3^ respectively (*p* = 0.02 and *p* = 0.022). Additionally, healthy perfused liver AD was significantly lower for TARE injection at a lobar position 50 ± 21.9 Gy versus 801 ± 24 Gy (*p* = 0.046). In this cohort, 2.7Fr microcatheter was used for 23 TARE injections mainly in multi-compartment treatments (8 out of 13) and when the injection was done at a lobar position (7 out 11) whereas the smaller 1.98/2.8Fr microcatheter, which was the most frequently used, was preferred for single-compartment treatments (33 out of 38) and selective approaches (34 out of 50 treatments at sub lobar positions). We found significant differences according to the type of microcatheter with significantly higher ^90^Y-Res with P-cat 138.7 ± 122.9 MBq versus 66.5 ± 57 MBq for AP-cat (*p* = 0.013). However, intended activity on pre-treatment ^99m^Tc-MAA SPECT/CT was also significantly higher with a mean value of 2198.3 ± 1320.9 MBq when P-cat was used versus 1464.2 ± 863.3 MBq for AP-cat (*p* = 0.013). Similarly, both perfused volume and tumor perfused volume were higher when P-cat was used when compared to the smaller one, 718 ± 566 cm^3^ versus 292 ± 273 cm^3^ and 328 ± 276 cm^3^ versus 144 ± 169 cm^3^, respectively (*p* = 0.003 and *p* = 0.014 respectively).

### Impact of ^90^Y-Res on ^99m^Tc-MAA SPECT/CT dosimetry

Looking at the impact of ^90^Y-Res on TARE dosimetry, we found a strong and positive correlation between ^90^Y-Res, the perfused volume (ρ = 0.651; *p* < 0.001), the tumor perfused volume (ρ = 0.608; *p* < 0.001) and the healthy tissue perfused volume (ρ = 0.737; *p* < 0.001) with higher ^90^Y-Res.

There were very strong positive association between AD measured in both perfused volume and perfused tumor volume calculated on pre-treatment ^99m^Tc-MAA SPECT/CT with the new values obtained after applying the previously mentioned “corrected activity” to pre-treatment ^99m^Tc-MAA SPECT/CT (Table 3). However, when comparing absorbed dose values between the pre-treatment ^99m^Tc-MAA SPECT/CT dosimetry and the ^99m^Tc-MAA SPECT/CT dosimetry using the “^90^Y-Res correction” there were no longer any significant difference (Table 3). The impact of ^90^Y-Res on personalized dosimetry was further investigated in a subgroup of HCC patients with borderline perfused tumor dosimetry on the initial pre-treatment ^99m^Tc-MAA SPECT/CT that is HCC patients with mean tumor AD within 205 Gy ± 10% for the perfused tumour volume (Table 4). In 1 out 5 patients with borderline but acceptable tumor AD on the pre-treatment ^99m^Tc-MAA SPECT/CT dosimetry the resulting tumor AD estimated after implementation of the “^90^Y-Res correction” was below the recommended threshold for HCC patients. This was interesting although a ^90^Y-PET/CT post-TARE imaging of the patient would have been useful to confirm this result.

**Table 3:** Comparison of TARE dosimetry as a function of ^90^Y-glass microspheres activity for each perfused volume; * *t*-test for dependent samples and ** Spearman correlation between AD value calculated on pre-treatment ^99m^Tc-MAA SPECT/CT with the ordered ^90^Y-microspheres activity ^1^ and a retrospective comparison with ^99m^Tc-MAA SPECT/CT when applying the “^90^Y-Res correction” ^2^ obtained after subtraction of ^90^Y-Res activity to ^90^Y activity in the TheraSphere™ vial; AD = Absorbed dose

		Estimated AD	Mean in Gy	Range in Gy	*p* value *	Rho and *p* values **
^99m^Tc-MAA SPECT/CT	Perfused volume	Pre-treatment ^1^	335 ± 259	[64–1280]	–	–
		With “^90^Y-Res correction” ^2^	326 ± 256	[62–1313]	0.16	0.950; < 0.001
	Perfused tumor	Pre-treatment ^1^	477 ± 456	[135–2353]	–	–
		With “^90^Y-Res correction” ^2^	473 ± 483	[151–2412]	0.17	0.998; < 0.001
	Healthy perfused liver tissue	Pre-treatment ^1^	63 ± 32	[29–125]	–	–
		With “^90^Y-Res correction” ^2^	54 ± 33	[33–103]	0.82	0.998; < 0.001

**Table 4:** Comparison of TARE dosimetry in HCC patients with borderline perfused tumor dosimetry (AD of 205 Gy ± 10%) on on pre-treatment ^99m^Tc-MAA SPECT/CT with the ordered ^90^Y-microspheres activity ^1^ and a retrospective comparison with ^99m^Tc-MAA SPECT/CT when applying the “^90^Y-Res correction” ^2^ obtained after subtraction of ^90^Y-Res activity to ^90^Y activity in the TheraSphere™ vial; in bold underlined tumor dosimetry with resulting estimated AD below the recommended threshold of 205 Gy for HCC tumor after the retrospective subtraction of ^90^Y-Res activity to the initial ^99m^Tc-MAA SPECT/CT; AD = Absorbed dose

HCC patients	Treatment type	Tumor perfused volume in cm^3^	Estimated AD in Gy on ^99m^Tc-MAA SPECT/CT		^90^Y-Res
			Pre-treatment ^1^	With “^90^Y-Res correction” ^2^	Absolute value in MBq
Patient 1	Multicompartment	57	226	216	187.2
Patient 2	Multicompartment	114	194	200	74.9
Patient 3	Segmentectomy	3	216	236	106.3
Patient 4	Segmentectomy	363	223	220	157.3
Patient 5	Multicompartment	96	216	**200**	230.3

## Discussion

Personalized dosimetry became the major key element in ^90^Y-microspheres TARE. Moreover, for an accurate calculation of absorbed doses, a precise measurement of the actual administered activity injected to the patient is essential. In our study, we reported a mean ^90^Y-Res below 5% (4.8 ± 3.5%, range 0.2–13.7) of the planned administered activity. This was slightly higher than the value reported by Drescher et al. (3.4 ± 1.7%, range 0.9–8.8%), which might be explained by the larger number of injections (n = 61) in our study when compared to their study (n = 18) (Drescher et al. 2020). ^90^Y-Res was strongly correlated to the activity in the sealed TheraSphere™ vial which might require optimizing some technical aspect of TARE such as a more thorough flushing of the TARE delivery system when higher activity is injected or additional controls of the delivery system using a survey meter during the procedure to reduce ^90^Y-Res. These aspects were not investigated in this work but certainly deserve further investigations. We did also find significant differences in the values of ^90^Y-Res according to the injection selectivity during TARE or the type of microcatheter used. These 2 parameters were also associated with significant differences in sizes for perfused volume and perfused tumor volume. There were also significant differences in the activity of the TheraSphere™ vial before TARE, indicating once again that this was the main factor associated with ^90^Y-Res. In cases of high ^90^Y-Res, the resulting tumor absorbed dose might be lower than predicted on pre-treatment ^99m^Tc-MAA SPECT/CT. This finding is relevant, especially in patients with borderline tumor dosimetry meaning patients with tumor predicted dosimetry equal or very close to the expected efficacy threshold on ^99m^Tc-MAA SPECT/CT. In routine practice a waste fraction of approximately 2% of the TheraSphere™ ordered activity is expected, hence a difference between the planned and the delivered activity should be expected. Nonetheless, in our series the difference was significantly higher. Indeed, though we found a mean ^90^Y-Res of 93.1 ± 94.6 MBq that is 4.8 ± 3.5% of the injected activity, on average 96.7 ± 72.3 MBq that is 5.8% ± 3.7 more activity was measured in the TheraSphere™ vial before the procedure in comparison to the planned therapeutic activity determined on the pre-treatment ^99m^Tc-MAA SPECT/CT. This might be related to our fixed days for the TARE procedure and subsequently the calibration date of the TheraSphere™ vial. Additionally, as we found that ^90^Y-Res was significantly correlated to the TheraSphere™ vial activity, a higher ^90^Y-Res should be expected in patients for which a higher ^90^Y-microspheres activity was ordered and it might be beneficial to adjust the ordered activity with at least 5% additional ^90^Y-microspheres activity for those patients. Nonetheless, though this finding was interesting, no significant difference for absorbed dose was found between the planned therapeutic activity and after the “^90^Y-Res correction” was applied, thus limiting the impact of ^90^Y-Res on perfused tumor and healthy perfused liver tissue absorbed dose. However, this was not the case for HCC patients for whom tumor dosimetry was already borderline on pre-treatment ^99m^Tc-MAA SPECT/CT as reported for 2 out of 22 HCC patients in this cohort that ultimately received a perfused tumor AD below the recommended 205 Gy threshold (Garin et al. 2021). Those results were an estimation with the “corrected activity” reported on pre-treatment ^99m^Tc-MAA SPECT/CT for each patients and a more accurate measurement of the final tumor AD with patients’ post-TARE ^90^Y-PET/CT should be done in addition to ^90^Y-Res measurement but it was not possible in our institution at the time ouf this study. Nonetheless, special attention should be given to an optimal flushing of the injection system especially when the activity in the TheraSphere™ vial is equal or slightly less than the planned activity on the initial pre-treatment ^99m^Tc-MAA SPECT/CT in patients with borderline tumor dosimetry. Indeed, it might prevent the recommended threshold of 205 Gy for the index lesions to be reached in these patients as an average residual activity of 5% could be expected. We did not explore the dosimetry aspect of the liver tumors outside of HCC due to the current lack of strong consensus on those tumors’ dosimetry and to the relatively smaller number of patients with cholangiocarcinoma and the various histology type of liver metastases.

The accurate calculation of post-treatment residual activity has been reported in the literature (Drescher et al. 2020; Ebbers et al. 2020). Yet, there is currently no consensus on the systematic measurement of the residual activity of ^90^Y-glass microspheres in patients undergoing TARE (Salem et al. 2023; Lambert et al. 2010; Garin et al. 2016). Additionally, though MIRD equations for doses calculation estimates the injected activity corrected for possible error factors it does not require an accurate measurement of post-treatment residual activity (Gulec et al. 2006; Bolch et al. 2009). However, an estimation of the final activity delivered to the patient during TARE has been recommended by The American association of physicists (Dezarn et al. 2011). The use of a dose calibrator was favored in comparison to a survey meter to measure post-TARE residual activity that should subsequently be subtracted to the patient’s planned injected activity to refine the final dosimetry (Dezarn et al. 2011). Chiesa et al. also included in their calculation for A_voxel_ (^90^Y), a correction of the activity injected in the liver A_liver_(^90^Y) as a function of the lung shunt fraction and the residual activity in the vial though the determination of the latter is not clear and do not account for the residual activity in the delivery tubing (Chiesa et al. 2015). In TARE with ^90^Y-resin microspheres, it is strongly recommended to estimate the post-procedure residual activity present in the vial and delivery system, though the methodology to achieve this measurement was not specified. A survey, a dose calibrator or imaging (^90^Y-PET/CT or ^90^Y-bremsstrahlung emission CT) were all available (Levillain et al. 2021). In our study, we chose to measure the residual activity in the vial and the delivery tubing including the microcatheter in a sealed plastic waste container for radioprotection purposes. We avoided disconnecting the delivery tubing after TARE as it might have led to additional exposure for the operators and an increased risk of radioactive contamination of the angiography suite. Additionally, although it would have been interesting to have a more precise assessment of the residual activity in the microcatheter independently of the rest of the delivery tubing to assess its impact more precisely in our study, it has already been reported in the literature. Drescher et al. showed that most of the residual activity measured using the activimeter or ^90^Y-PET/CT after TARE injection was contained in the microcatheter and connector (Drescher et al. 2020). However, due to discrepancies reported in the literature between ^99m^Tc-MAA SPECT/CT prediction and actual distribution of ^90^Y microspheres resin or glass, when available ^90^Y-PET/CT with time-of-flight is optimal to more accurately define the actual tumor dosimetry (Chiesa et al. 2021). Unfortunately, due to reimbursement issues in Switzerland at the time of the study, ^90^Y-PET/CT post-TARE was not systematically available for the patient or to measure ^90^Y-Res. We chose not to explore ^90^Y-bremsstrahlung SPECT/CT imaging in this study as it was not quantitative and the vendor protocol used in our machine in routine practice uses an algorithm that adjusts reconstruction parameters to obtain an image with an acceptable noise signal, hence those parameters might vary depending on the patient. However, interesting results were reported in the literature related to the use of SPECT/imaging post-TARE (Kim et al. 2021). Nonetheless, further investigations comparing those results with ^90^Y-PET/CT post-TARE should be done and if confirm could benefit other institution without routine ^90^Y-PET/CT post-TARE available (Kim et al. 2021). Instead, we use a survey dose-rate and ^90^Y-PET/CT is reserved to cases where post-treatment residual activity is higher than expected. We currently use a threshold of ≥ 10% of the dose-rate measured from the TheraSphere™ vial before the injection. The comparison of ^90^Y-Res measurements between the survey meter and the ^90^Y-PET/CT was not within the spectrum of this study and has been reported in the literature (Dezarn and Kennedy 2007). Indeed, the use of a survey dose-rate meter to assess residual activity post-TARE has shown consistent results with dose calibrator measurements in the setting of ^90^Y-resin microspheres whereas it was not the case for ^90^Y-glass microspheres (Ebbers et al. 2020; Dezarn and Kennedy 2007). Nonetheless, the use of a survey dose-rate meter remains essential in routine practice and the use of ^90^Y-PET/CT scan diminish the risk of contamination and overall radiation exposure for the workers in comparison to the use of a dose calibrator (Ebbers et al. 2020). In our institution, the measurement of ^90^Y-Res using ^90^Y-PET/CT is also done in selected cases if the patients require an additional ^90^Y-PET/CT scan after conventional ^90^Y-bremsstrahlung emission to precisely define tumor absorbed dose in particular cases such as significant differences in ^90^Y-microspheres distribution in the perfused volume in comparison to pre-treatment ^99m^Tc-MAA SPECT/CT.

The impact of ^90^Y-Res on calculation of AD of the perfused tumor volume might have been underestimated in our cohort due to the clear imbalance between multi-compartment and single-compartment treatment. Indeed, there is a proportion of 79% radiation segmentectomies in our study for which the mean tumor absorbed dose was 548 Gy that is more than 2 times higher in comparison to the 223 Gy AD in multi-compartment treatment. Thus, in the case radiation segmentectomy treatments as tumor AD was very high on ^99m^Tc-MAA SPECT/CT a ^90^Y-Res of 5% will not have a significant impact on tumor absorbed dose according to the currently recommended threshold of 205 Gy for tumor AD in HCC patients who represent the vast majority of our population (Garin et al. 2021). AD was less likely to be affected if the activity after the “^90^Y-Res correction” was slightly lower than planned. Indeed, optimizing perfused tumor AD while limiting the volume of healthy perfused liver tissue within the perfused volume prompt to a radiation segmentectomy approach. Therefore, TARE with multiple injections to improve tumor targeting was preferred in our institution with at least 2 different arterial positions in 19/41 TARE procedures (49%). Additionally, in this population, 6 patients had multiple TARE procedures usually in the setting of very large or bi-lobar tumor infiltration.

A potential limitation in our study was that the influence of the day of the TARE procedure with respect to the date of the calibrated vial activity which was not investigated. The calibration date affects the number of microspheres for a matched activity, hence microsphere specific activity (Spreafico et al. 2014). In our institution we had during the time of this study significantly higher ^90^Y activity delivered on the day of the TARE procedure compared to the activity defined on the pre-treatment ^99m^Tc-MAA SPECT/CT which most likely related to our fixed days for the TARE procedure that is Thursdays and Fridays, which would automatically impact the dose calibration date for the manufacturer. Thus, as previously mentioned when we subtracted the ^90^Y-res to the activity measured on the TheraSphere™ vial using an activimeter calibrated to the national standard the resulting “the corrected activity” was similar to the planned activity on the pre-treatment ^99m^Tc-MAA SPECT/CT limiting the impact of ^90^Y-res in our institution but this might not be the case in other institutions without fixed days for the TARE procedure.

Another important issue affecting the accuracy of the estimated AD in target tissues was pointed out by Gnesin et al. (2023) and corroborated by Auditore et al. using Monte-Carlo simulation, Auditore et al. (2023). They measured discrepancies between the calibrated ^90^Y vial activities stated by the vendors and the measured activity of the same vials obtained with quantitative ^90^Y-PET/CT (Auditore et al. 2023). For the case of ^90^Y-glass microspheres, an overestimate of the vial activity by a 20% have been reported compared to PET assessment and ^90^Y Chloride calibrated vial. In absence of new dosimetry results at the time of analysis considering the reported discrepancies, in the present study we neglected this effect that has a direct impact in absolute quantification of the vial and though it might also affect the relative percent difference when compared to the TheraSphere™ vial measured before TARE using the activimeter it would not affect the absolute value in MBq of ^90^Y-Res measured using ^90^Y-PET/CT which showed consistent and significant differences in our study. Additionally, in this study ^90^Y-PET/CT provided with quantitative assessment of the ^90^Y activity in the acquired field of view based on its calibration with the ^18^F. The quantification of ^90^Y it is obtained from the ^18^F quantification based on the knowledge of the radioscopes half-lives and beta + particle emission yield. Our PET/CT had a quantitative accuracy of ~ 5% in respect to the ^18^F. It is expected to have a similar accuracy for the ^90^Y. The expected quantitative accuracy for the ^90^Y is also corroborated by recent publications (Gnesin et al. 2023; Auditore et al. 2023) where the ^90^Y-PET activity measured in vials containing a homogenous activity concentration of ^90^Y was in a ~ 5% agreement with the calibrated activity declared by the vial vendor. We should also mention that the measurement of the ^90^Y residual activity was measured with three independent methods, that was ^90^Y-PET/CT, a survey dose-rate and an activimeter and the results of this study will soon be published.

Despite the previously mentioned limitations we thought this study to be of value, in this era of personalized dosimetry, the accurate calculation of the perfused tumor AD is essential as it is associated with tumor response and survival outcomes as reported in the recent literature (Garin et al. 2021; Lam et al. 2022; Salem et al. 2021). Thus, prompting the implementing of a routine procedure for measuring post-TARE residual activity after each procedure to quickly determine which patient might benefit from an additional ^90^Y-PET/CT scan to verify if the target lesion received the recommended absorbed dose despite a higher residual ^90^Y-micropheres activity after the procedure. This aspect is particularly relevant in nuclear medicine services with limited availability of ^90^Y-PET/CT which restrict the possibility to systematically calculated the accurate perfused tumor AD though its significant association with objective response rate has been demonstrated in a prospective trial of HCC patients (Chan et al. 2018). Additionally, to consolidate the importance of personalized dosimetry, more technical developments are expected to be implemented in the future, such as 3D voxel dosimetry that could improve the quantification of ^90^Y-microspheres distribution (Balagopal and Kappadath 2018). Moreover, it seems reasonable that a standardization of the measurement of the residual activity should also be implemented in routine practice (Veenstra et al. 2022).

## Conclusion

Our study reported original results on the measurement of ^90^Y-Res using ^90^Y-PET/CT after TARE with an average of 5% residual ^90^Y-glass microspheres activity not delivered to the patient. The injection selectivity and the type of microcatheter were significantly associated with ^90^Y-Res, but the strongest association was found with the injected activity suggesting that the higher injected activity the higher ^90^Y-Res value and a standardization of its measurement should be implemented in clinical practice. Therefore, when a high ^90^Y-Res is suspected on the survey meter, additional investigations such as a ^90^Y-PET/CT scan of ^90^Y-Res and a dedicated post-TARE ^90^Y-PET/CT imaging of the patient’s treatment site should be done if possible for a more accurate tumor dosimetry accounting for the ^90^Y-microspheres activity ultimately injected to the patient to assess if the target lesion did receive the recommended AD. This is especially important in HCC patients for whom TARE is planned as a multicompartment treatment with a borderline perfused tumor or healthy perfused tissue dosimetry on the initial pre-treatment ^99m^Tc-MAA SPECT/CT.

## Data Availability

The datasets used and/or analysed during the current study are available from the corresponding author on reasonable request.

## Abbreviations

^90^Y-Res: ^90^Y-glass microspheres residue
^99m^Tc-MAA SPECT/CT: ^99M^Tc-macroaggregated albumin single-photon emission computerized tomography/ computerized tomography
AD: Absorbed dose
AP-cat: ASAHI Masters Parkway Soft 1.98/2.8 F microcatheter
HCC: Hepatocellular carcinoma
P-cat: Progreat 2.7Fr. microcatheter
TARE: Transarterial radio-embolization
